# Curcumin nanocrystals ameliorate ferroptosis of diabetic nephropathy through glutathione peroxidase 4

**DOI:** 10.3389/fphar.2024.1508312

**Published:** 2025-01-06

**Authors:** Mengjiao Xue, Yiwei Tian, Hua Zhang, Shijie Dai, Yangsheng Wu, Juan Jin, Jian Chen

**Affiliations:** ^1^ School of Clinical Medicine, Hangzhou Medical College, Hangzhou, China; ^2^ Department of Nephrology, the First Affiliated Hospital of Zhejiang Chinese Medical University (Zhejiang Provincial Hospital of Chinese Medicine), Hangzhou, China; ^3^ School of Pharmacy, Shanghai Jiaotong University, Shanghai, China; ^4^ College of Life Science, Academy of Chinese Medical Sciences, Zhejiang Chinese Medical University, Hangzhou, China

**Keywords:** curcumin, nanocrystals, ferroptosis, diabetic nephropathy, GPX4

## Abstract

**Objective:**

The aim of this study was to investigate the effect of curcumin nanocrystals (Cur-NCs) on ferroptosis in high-glucose (HG)-induced HK-2 cells and streptozotocin (STZ)-induced diabetic nephropathy model (DN) rats. The purpose is to determine whether Cur NCs can become a promising treatment option for diabetes nephropathy by reducing ferroptosis.

**Methods:**

Cur-NCs were prepared using microfluidic technology and studied using dynamic light scattering and transmission electron microscopy. HK-2 cells were treated with 30 mM HG to create a renal tubule damage cell model. Then, cell viability was evaluated in HK-2 cells treated with varying concentrations of Cur-NCs (0.23, 0.47, 0.94, 1.87, 3.75, 7.5, 15, and 30 μg/mL) using Cell Counting Kit-8 (CCK-8). Furthermore, *in vivo* experiments were carried out to investigate the roles of Cur-NCs in STZ-induced DN rats.

**Results:**

The results showed that HG treatment greatly enhanced the levels of LDH, MDA, Iron, lipid ROS, apoptosis, NCOA4, TFR-1, while decreasing the expression of GSH, GPX4, SLC7A11, and FTH-1. These effects induced by HG could be attenuated by Cur-NCs. Cur-NCs also reduced the HG-induced decrease in cell viability, as well as the increase in lipid ROS and cell apoptosis, however erastin could inhibit their effects. Furthermore, the *in vivo* results showed that Cur-NCs reduced ferroptosis and inhibited renal damage in DN rats.

**Conclusion:**

This study demonstrates that Cur-NCs can significantly attenuate ferroptosis in a STZ-induced renal damage model by recovering GPX4, implying that Cur-NCs may be a promising therapy option for DN.

## Introduction

The primary cause of end-stage renal disease (ESRD) worldwide is diabetes nephropathy (DN), which not only seriously compromises patient quality of life but also places a significant financial and social strain on the world’s healthcare system (Samsu, 2021; Hu et al., 2023). Its prevalence rate is rising annually, particularly in the population with a high incidence of diabetes, making it an urgent public health concern. Worldwide, an estimated 537 million people have been diagnosed with diabetes (Sun et al., 2022). Up to 30%–40% of people in these sizable diabetes patient groups are predicted to develop DN (Selby and Taal, 2020). The pathogenesis of DN is complex and multifaceted, including multiple factors such as metabolic abnormalities, hemodynamic disorders, and inflammatory responses (Lin et al., 2018; Chen et al., 2022a). DN is primarily caused by chronic hyperglycemia, which sets off a cascade of events in the kidneys that include aberrant activation of protein kinase C (PKC) subtypes, build-up of advanced glycation end products (AGEs), and excessive production of reactive oxygen species (ROS) (Tanase et al., 2022; Feng et al., 2023). These molecular level changes gradually erode kidney health, leading to dysfunction of renal endothelial cells, apoptosis of podocytes, mesangial dilation, ultimately resulting in glomerulosclerosis and tubulointerstitial fibrosis (Tanase et al., 2022; Yao et al., 2022). At the same time, renal hemodynamic dysregulation characterized by glomerular filtration and hypertension further exacerbates kidney damage (Tuttle et al., 2022). Furthermore, the kidneys’ inflammatory response and fibrosis process are made worse by the involvement of several pro-inflammatory cytokines, including interleukin (IL), tumor necrosis factor-alpha (TNF-α), and transforming growth factor-beta (TGF-β) (Wang et al., 2021b; Ahmed et al., 2022). While significant progress has been made in exploring the pathophysiological mechanisms of DN, there is still a dearth of efficient treatments to slow the disease’s progression. This necessitates further research to fully understand its complex mechanisms and create innovative therapies that can effectively intervene and delay the devastating effects of DN.

In recent years, the growing idea of ferroptosis, a new type of controlled cell death marked by iron-dependent lipid peroxidation, has received a lot of attention in the field of DN (Li et al., 2023b; Mengstie et al., 2023). The disorders of iron homeostasis and lipid metabolism are the key driving factors of DN pathogenesis, which would further trigger and exacerbate ferroptosis. In various studies, quite a number of molecular pathways have been confirmed to be implicated in it, including the iron-regulatory proteins, the phospholipid metabolism pathway involving the enzyme acyl-CoA synthetase long-chain family member 4 (ACSL4), and the glutathione peroxidase 4 (GPX4)-regulated antioxidant defense system (Li et al., 2022a; Wu et al., 2023). Furthermore, diabetic conditions promote oxidative stress and inflammation, which may amplify ferroptotic cell death in renal cells (Shen et al., 2022). Thus, investigating the function of ferroptosis in DN is expected to provide new therapeutic targets for minimizing renal damage and maintaining renal function in diabetics. However, further research into the specific biochemical processes involved and the development of appropriate therapeutics are still needed to fully realize the potential of addressing ferroptosis in the treatment of diabetic nephropathy.

Traditional Chinese medicines (TCM) provide a holistic approach to attenuate renal damage and delay disease progression with antioxidant, anti-inflammatory and antifibrotic properties (Chen et al., 2023; Liang et al., 2024). For example, salvinorin B and tanshinone IIA synergistically attenuate early DN by upregulating the PI3K/Akt pathway and downregulating the NF-κB signaling pathway (Xu et al., 2024b). In addition, mullein isoflavone extracts have anti-ferroptosis sagging effects by downregulating lipid ROS in DN (Huang et al., 2022). Ginkgolide B ameliorates DN oxidative stress and ferroptosis by inhibiting GPX4 ubiquitination (Chen et al., 2022b). Therefore, researchers are increasingly focusing on ferroptosis-suppressive drugs as they show good potential to treat human diseases.

In contemporary scientific research, the exploration of novel therapeutic approaches for DN has led to a growing interest in the application of curcumin as a potential treatment modality (Zhu et al., 2022). A naturally occurring polyphenol chemical, curcumin, is extracted from the rhizome of Curcuma longa L. With its wide range of pharmaco logical characteristics, such as its anti-inflammatory, anti-fibrosis, and antioxidant activities, curcumin has a lot of promise for DN intervention (Wang et al., 2023; Yang et al., 2024). Accumulating evidence has shown that curcumin attenuated apoptosis of podocyte cells and accelerated cell autophagy in DN via regulating Beclin1/UVRAG/Bcl2 pathway (Zhang et al., 2020). Curcumin also decreases oxidative stress by keeping the Nrf2 pathway in a steady state, dramatically improving retinal damage in diabetes (Xie et al., 2021). Furthermore, curcumin can suppress ERK/JNK phosphorylation in insulin resistance cells generated by high glucose (HG) and promote the PI3K-AKT-GSK3B signaling pathway, hence increasing insulin sensitivity, providing a novel avenue for targeted diabetes treatment (Hussain et al., 2022). These results suggest that curcumin could be a new therapy option for diabetic nephropathy.

Curcumin’s fast metabolism, low bioavailability, and poor water solubility, however, make it difficult to translate clinically (Chen et al., 2022b). Fortunately, the rapid advancement of nanotechnology has created more opportunities for the clinical application of curcumin. Curcumin nanocrystals (Cur-NCs) meticulously created by nanotechnology not only considerably improve the solubility and bioavailability of the medicine (Wang et al., 2021a), but also lengthen its retention time in the body, significantly increasing the therapeutic effect (Huang et al., 2023; Wang et al., 2023). This novel achievement has broadened the application possibilities for Cur-NCs in the treatment of DN. However, our current exploring of Cur-NCs is insufficient, necessitating more in-depth studies *in vitro* and *in vivo* to further elucidate their potential mechanism of action, optimize the formulation parameters of their clinical translation, so that they can function as effectively as possible in therapeutic practice.

## Materials and methods

### Preparation of Cur-NCs

A total of 40 mg of curcumin (HXJHS20200710, purity: 98%, Xi’an Haoxuan Biotech Co., Ltd., China) was dissolved in 4 mL of acetone to form a 10 mg/mL solution and then passed through 0.22 μm microporous filter to obtain the organic phase solution. Polyvinylpyrrolidone K30 (PVP-K30, 50 mg, Shanghai yuanye Bio-Technology Co., Ltd., China) was accurately weighed and dissolved in 10 mL of water to obtain the aqueous phase solution. The organic and aqueous phase solutions were fed into a stainless steel microfluidic chip with a split-and-recombine (SAR) structure by MPE-L2 microfluidic instrument (Suzhou Aitesen Pharmaceutical Equipment CO., Ltd., China) through a syringe pump, and the flow rate of the two phases was controlled by a syringe pump connected to the system at 9 and 1 mL/min, respectively. After the system was stabilized, the curcumin suspension was collected with a beaker at the outlet and freeze-dried to obtain Cur-NCs powder.

### Particle size, Zeta potential, and morphology

The particle size and zeta potential of Cur-NCs (Zheng et al., 2024) were detected in purified water by Zetasizer Nano ZS (Malvern Instruments, United States). For morphological examination, Cur-NCs were mounted on 300-mesh Cu grids and negatively stained with 1% uranyl acetate for 2 min. Transmission electron microscope (TEM) images were taken using a Talo L120C G2 TEM (United States) operating at 200 keV. The average length and width of 200 randomly selected Cur-NCs were identified from the TEM images using ImageJ software (v1.53, NIH, United States).

### Cell culture and treatment

Human renal proximal tubular epithelial cells (HK-2) were obtained from the iCell Bioscience Inc (Shanghai, China). The cells were cultured in Dulbecco’s Modified Eagle Medium/Nutrient Mixture F-12 (11330032, Gibco, United States) supplemented with 10% fetal bovine serum (A5669701, Gibco, United States) in a humidified incubator at 37°C with 5% CO_2_. To induce HG conditions, HK-2 cells were treated with 30 mM D-glucose, while the normal glucose (control) group was exposed to 5.5 mM D-glucose and 24.5 mM mannitol for 48 h. HK-2 cells were exposed to control or HG, with or without Cur-NCs (1.87, 3.75, 7.5 μg/mL), Ferrostatin-1 (1 μM, S81461, Shanghai yuanye Bio-Technology Co., Ltd., China), Erastin (35 μM, S80804, Shanghai yuanye Bio-Technology Co., Ltd., China) treatment alone or in combination for the specified time (0 h, 12 h, 24 h, or 48 h).

### siRNA transfection

When HK-2 cells were seeded in 6-well plates at 80% confluence. Then, Lipofectamine™ 3,000 (L3000015, Invitrogen, United States) was diluted with Opti-MEM medium and mixed thoroughly. The short interfering RNAs (siRNAs) used were siRNA_GPX4_NC (5′-UUC​UCC​GAA​CGU​GUC​ACG​UTT-3′), siRNA_GPX4_#1 (5′-ACC​AAG​TTT​GGA​CAC​CGT​CTC​TCC​A-3′), siRNA_GPX4_#2 (5′-CAA​GTT​TGG​ACA​CCG​TCT​CTC​CAC​A-3′), and siRNA_GPX4_#3 (5′-ACA​CCG​TCT​CTC​CAC​AGT​TCC​TCA​T-3′). These siRNAs were also diluted with Opti-MEM medium in the same way and mixed thoroughly. Then, the diluted siRNAs and the diluted Lipofectamine™ 3,000 were mixed at a ratio of 1:1 at room temperature and incubated for 10 min. The original culture medium was removed, and the cells were washed twice with serum-free medium. Subsequently, fresh serum-free medium was added, and the complexes were added into the wells and evenly distributed. The cells were incubated for 4 h, and then the medium containing serum was replaced. After transfection for 48 h, the transfection efficiency was measured by using quantitative real-time PCR (qRT-PCR) and Western blotting assays.

### Cell counting kit-8 (CCK-8) assay

Cell viability was assessed using the CCK-8 assay according to the manufacturer’s instructions (C0039, Beyotime Biotechnology, China). Briefly, HK-2 cells were seeded in 96-well plates at a density of 5,000 cells per well and allowed to adhere overnight. Following treatment with various experimental conditions, 10 μL of CCK-8 solution was added to each well and incubated for 2 h at 37°C. The absorbance was measured at 450 nm using a microplate reader (Molecular Devices).

### Flow cytometry assay

ROS levels were quantified using flow cytometry analysis. After treatment with experimental conditions, the treated HK-2 cells were harvested and washed with phosphate-buffered saline (FG701-01, TransGen, China). Subsequently, cells were incubated with 10 μM 2′,7′-dichlorofluorescein diacetate (DCFH-DA) probe (S0033M, Beyotime Biotechnology, China) for 30 min at 37°C in the dark to detect intracellular ROS. Following incubation, cells were washed and resuspended in PBS. Flow cytometry analysis was performed using a BD Accuri™ C6 flow cytometer (BD Biosciences, United States), and ROS levels were quantified based on the fluorescence intensity of oxidized DCFH. Data were analyzed using Tree Star FlowJo software.

### TEM assay

TEM was employed to investigate the ultrastructural characteristics of HG-induced HK-2 cells treated with Cur-NCs or Fer-1. Before imaging, HK-2 cells were cultured in appropriate growth media and seeded onto carbon-coated electron microscopy grids. Following fixation with 2.5% glutaraldehyde in 0.1 M phosphate buffer (pH 7.4) for 2 h at room temperature, cells were post-fixed with 1% osmium tetroxide in the same buffer for 1 h. Dehydration was achieved through a graded series of ethanol solutions, and cells were then embedded in Epon resin. Ultrathin sections (70 nm) were cut using a diamond knife on an ultramicrotome and collected on copper grids. Sections were contrasted with uranyl acetate and lead citrate before examination under a TEM (H-7650, Hitachi, Japan) operating at an accelerating voltage of 120 kV. Images were captured using a CCD camera (Gatan Orius SC1000, United States) at various magnifications to assess cellular morphology and subcellular structures.

### Terminal deoxynucleotidyl transferase dUTP nick end labeling (Tunel) staining

Tunel staining (C1090, Beyotime Biotechnology, China) was performed to assess the apoptosis levels in HK-2 cells. Cultured HK-2 cells were subjected to various experimental conditions as appropriate and subsequently fixed with 4% paraformaldehyde in PBS for 15 min at room temperature. Following fixation, cells were permeabilized with 0.1% Triton X-100 in PBS for 5 min and then incubated with the 50 μL of Tunel reaction mixture containing terminal deoxynucleotidyl transferase enzyme and fluorescein-labeled dUTP for 1 h at 37°C in a humidified chamber. After Tunel staining, cells were counterstained with DAPI (4′,6-diamidino-2-phenylindole) for nuclear visualization. The stained cells were imaged using a fluorescence microscope (Nikon DS-Fi2, Japan) equipped with appropriate filter sets. Quantification of Tunel-positive cells was performed by counting labeled nuclei in randomly selected fields of view.

### Animals and treatment

All animal experiments were approved by the Institutional Animal Care and Use Committee of Zhejiang Center of Laboratory Animals (ZJCLA) (Approval number: ZJCLA-IACUC-20010530). A total of 42 6-week-old male Sprague-Dawley rats (Tu et al., 2019; Valdivielso et al., 2019; Noshahr et al., 2020; Ganugula et al., 2023) were obtained from Zhejiang Center of Laboratory Animals. Rats were housed at 22°C ± 2°C, a 12 h light-dark cycle, 50% ± 5% humidity, with free access to food and water. After acclimatization for 7 days, 6 rats were randomly assigned to the control group. Following overnight fasting, the rats were received a single intraperitoneal injection of streptozotocin (S0130, Sigma, Germany), which was dissolved in sodium citrate buffer solution (pH 4.0, R21466, Shanghai yuanye Bio-Technology Co., Ltd., China) at a dosage of 65 mg/kg (ALTamimi et al., 2021; Machado et al., 2022). Three days after the injection, the blood glucose levels were measured by using a blood glucose meter (Accu-Chek). Rats with fasting blood glucose levels exceeding 16.7 mmol/L for three consecutive days were diagnosed with diabetes (Supplementary Figure S1) and then included in the subsequent research. To investigate the therapeutic efficacy of Cur-NCs, DN rats were randomly assigned to five groups: Model, Cur-NCs low-dose, Cur-NCs middle-dose, Cur-NCs high-dose, and Fer-1 groups, with 6 rats in each group. Rats in the control and model groups were intraperitoneally injected with an equal volume of buffered saline solution daily; Rats in Fer-1 group received daily intraperitoneal injection of Fer-1 (2.5 μmol/kg, S81461, Shanghai yuanye Bio-Technology Co., Ltd., China); and rats in the low-dose group, medium-dose group, and high-dose group of Cur-NCs were intraperitoneally injected with 100, 200, and 400 μg/kg of Cur-NCs daily (Zheng et al., 2024), respectively. The rats were injected once daily for 8 weeks. Animal body weight were determined weekly until the end of the experiment. At the end of the experiment, all were housed in metabolic chambers for 24 h to collect urine samples and record urine volume. U-mAlb (Urinary microalbumin) and Ucrea (Urinary creatinine) levels in urine samples were detected using a biochemical analyzer (Hitachi 7020, Japan). After that, the rats were fasted overnight and anesthetized with isoflurane. Then blood was collected from the heart, and centrifuged at 3,000 rpm for 15 min to obtain the serum. The levels of TC, TG, LDL-C, HDL-C, BUN, and SCr in each group of rats were determined using a biochemical analyzer (Hitachi 7020, Japan). Based on the obtained data of urinary creatinine, plasma creatinine, urine volume and body weight, glomerular filtration rate (GFR) was calculated according to the formula “GFR = (Ucrea/SCr) * urine volume/body weight” (Gao et al., 2020; Zhao et al., 2022). Finally, the kidneys were removed immediately, weighed, and used to calculate the kidney/weight index (KWI). The freshly dissected kidneys were preserved at −80°C for subsequent experiments.

### Enzyme-linked immunosorbent assay (ELISA)

Levels of LDH (A020-2-2, Nanjing Jiancheng, China), MDA (MM-0436H, Jiangsu Meimian Industrial Co., Ltd., China), Iron (Fe^2+^, BC5310, Solarbio, China), and GSH (MM-0458H, Jiangsu Meimian Industrial Co., Ltd., China) in the cultured HK-2 cells, as well as the levels of MDA, SOD, Cat, and GSH (MM-0385R2, MM-0386R1, MM-20447R1, MM-0602R1; Jiangsu Meimian Industrial Co., Ltd., China) in kidney tissue samples were measured using the commercial kits according to the manufacturer’s instructions.

### Renal histological analysis

Histological analyses were performed on kidney tissue samples using a combination of hematoxylin and eosin (H&E), Masson’s trichrome, and Periodic Acid-Schiff (PAS) staining techniques. Briefly, kidney tissues were fixed in 10% paraformaldehyde for 24 h, embedded in paraffin, and sectioned into 5-µm-thick slices. For H&E staining, tissue sections were deparaffinized, rehydrated, and stained with Hematoxylin (H3136, Sigma, Germany) for 5 min followed by Eosin (E4009, Sigma, Germany) for 2 min. Masson’s trichrome staining was conducted to assess collagen deposition and fibrosis, with tissue sections subjected to Weigert’s iron hematoxylin (R20387, Shanghai yuanye Bio-Technology Co., Ltd., China), Ponceau S acid fuchsin stain (71019360, 71033761, Sinopharm Chemical Reagent Co., Ltd., China), and Aniline blue (71003644, Sinopharm Chemical Reagent Co., Ltd., China) staining solutions. For PAS staining to evaluate glycogen and basement membranes, tissue sections were oxidized with periodic acid (C0142S, Beyotime Biotechnology, China), treated with Schiff’s reagent (C0142S, Beyotime Biotechnology, China), and counterstained with Hematoxylin (Bry-0001-01, Runner bio, China). Stained sections were examined under a light microscope (×400; Nikon, Japan).

### qRT-PCR assay

Total RNA was extracted from the treated HK-2 cells and kidney samples using an EZ-10 Total RNA Mini-Preps Kit (B618583-0100, Sangon, China) according to the manufacturer’s instructions. The quantity and quality of RNA were determined using a spectrophotometer. Complementary DNA (cDNA) was synthesized from the purified RNA using a high-capacity cDNA reverse transcription kit (CW2569, CoWin Biotech Co., Ltd., China). qRT-PCR was performed on a real-time PCR system (LightCycler^®^ 96, Roche, Switzerland) with specific primers designed for the target genes and a SYBR Green PCR master mix (11201ES08, YeasenBiotechnology Co., Ltd., China). The detailed information of the related primer sequences is presented in Supplementary Table S1. The amplification conditions included an initial denaturation step at 95°C for 10 min, followed by 40 cycles of denaturation at 95°C for 15 s and annealing/extension at 60°C for 1 min. The relative expression levels of the target genes were calculated using the 2^−ΔΔCt^ method.

### Western blot analysis

Western blot analysis was conducted to assess the protein expression levels of GPX4, NCOA4, FTH-1, TFR-1, and SLC7A11 in both treated HK-2 cells and kidney tissues. Total protein was extracted from the treated HK-2 cells and kidney samples using a RIPA buffer (P0013B, Beyotime Biotechnology, China) supplemented with protease and phosphatase inhibitors (CW2200S, CoWin Biotech Co., Ltd., China). Protein concentrations were determined using a Bradford protein assay kit (P0012, Beyotime Biotechnology, China). Equal amounts of protein were resolved on sodium dodecyl sulfate-polyacrylamide gel electrophoresis (SDS-PAGE) and transferred onto polyvinylidene fluoride (PVDF) membranes. Membranes were blocked with 5% non-fat milk in Tris-buffered saline with 0.1% Tween-20 (TBST) for 1 h at room temperature and then incubated overnight at 4°C with primary antibodies specific for GPX4 (1:1,000, ab125066, Abcam, Britain), NCOA4 (1:1,000, ab86707, Abcam, Britain), FTH-1 (1:1,000, ab75973, Abcam, Britain), TFR-1 (1:1,000, ab214039, Abcam, Britain), SLC7A11 (1:1,000, ab175186, Abcam, Britain), and β-actin (1:10,000, AF7018, Affinity, United States). Following primary antibody incubation, membranes were washed with TBST and incubated with appropriate secondary antibodies conjugated to horseradish peroxidase (HRP) for 1 h at room temperature. Protein bands were visualized using an enhanced chemiluminescence (ECL) detection system, and densitometric analysis was performed using ImageJ software. β-actin was used as a loading control to normalize protein expression levels.

### Statistical analysis

All results are shown as the mean ± SD. A significant difference was analyzed using SPSS software (Version 22.0, IBM Corp., United States). Statistical analysis was carried out using two-paired-tailed *t-tests* or one-way analysis of variance (ANOVA) with Tukey’s *post hoc* test. *p < 0.05* was considered statistically significant.

## Results

### Cur-NCs ameliorated the cell viability of HG-induced HK-2 cells

**FIGURE 1:**
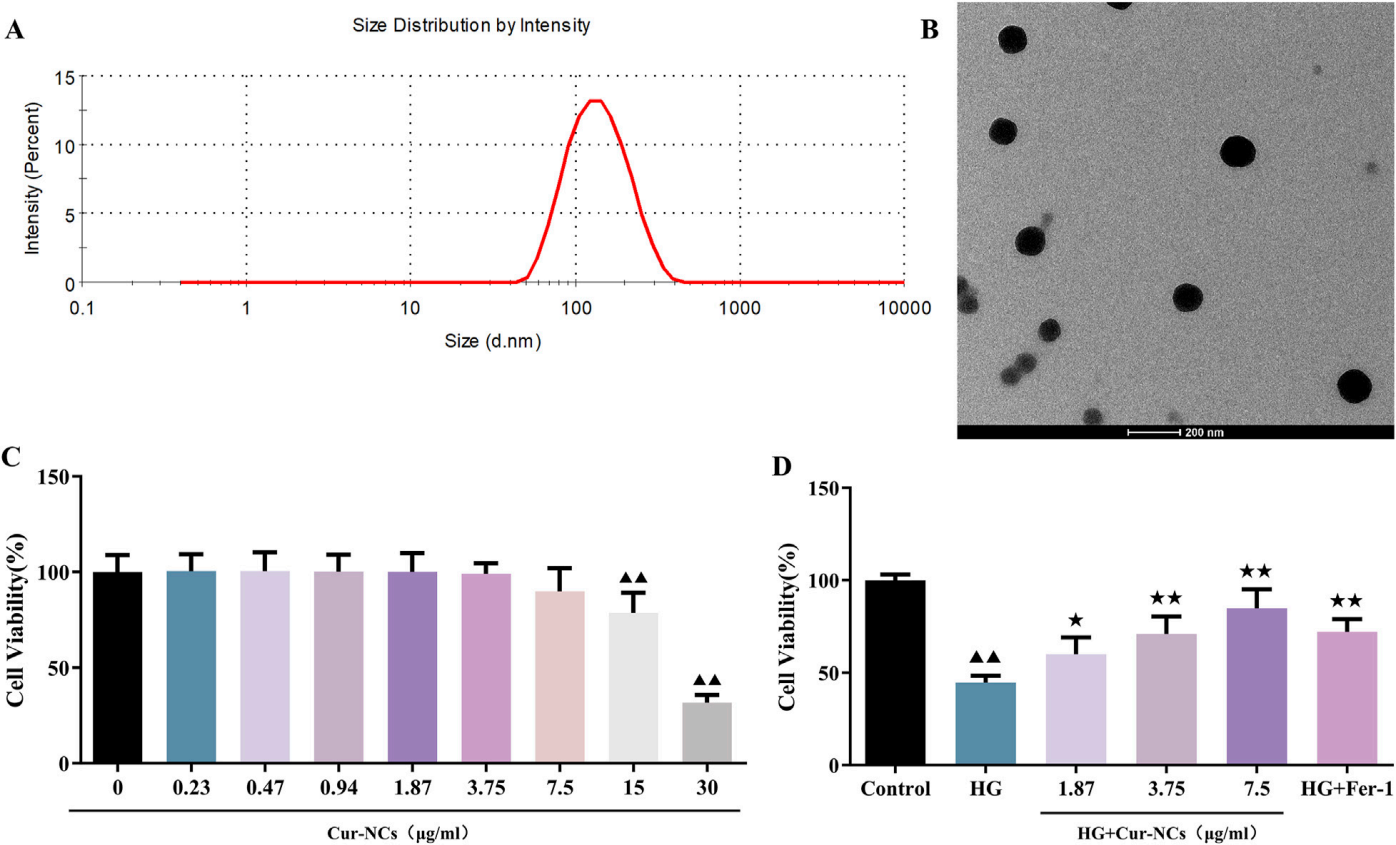
Physicochemical characterization of Cur-NCs and effect of Cur-NCs on HK-2 cell viability. **(A)** Mean particle size detected by DLS (Z-average, nm) and zeta potential (mV) of Cur-NCs. **(B)** Representative TEM images of Cur-NCs. **(C)** HK-2 cell viability was assessed post-treatment with different concentrations of Cur-NCs. **(D)** The CCK-8 assay was conducted to measure the cell viability of HG-induced HK-2 cells with Cur-NCs treatment. ^▲▲^ *p < 0.01* vs. the control group; ^★^ *p <* 0.05, ^★★^ *p <* 0.01 vs. the HG group.

Cur-NCs had an average size of 122.0 ± 2.464 nm, a PDI of 0.167 ± 0.024, and a zeta potential of −17.36 ± 2.38 mV (Figure 1A). Concurrently, TEM showed that the Cur-NCs were circular-shaped with a uniform size (Figure 1B). The CCK-8 test was used to measure cell viability in order to ascertain the effects of various doses of Cur-NCs on HK-2 cells. The viability of HK-2 cells did not significantly decrease when the concentration of Cur-NCs was increased to 15 μg/mL, as Figure 1C illustrates. Furthermore, we discovered that the viability of HG-induced HK-2 cells was considerably increased by dosages of 1.87, 3.75 and 7.5 μg/mL of Cur-NCs and Fer-1 (Figure 1D). Therefore, 1.87, 3.75 and 7.5 μg/mL of Cur-NCs were selected for subsequent treatment.

### Cur-NCs ameliorated oxidative damage in HG-induced HK-2 cells

**FIGURE 2:**
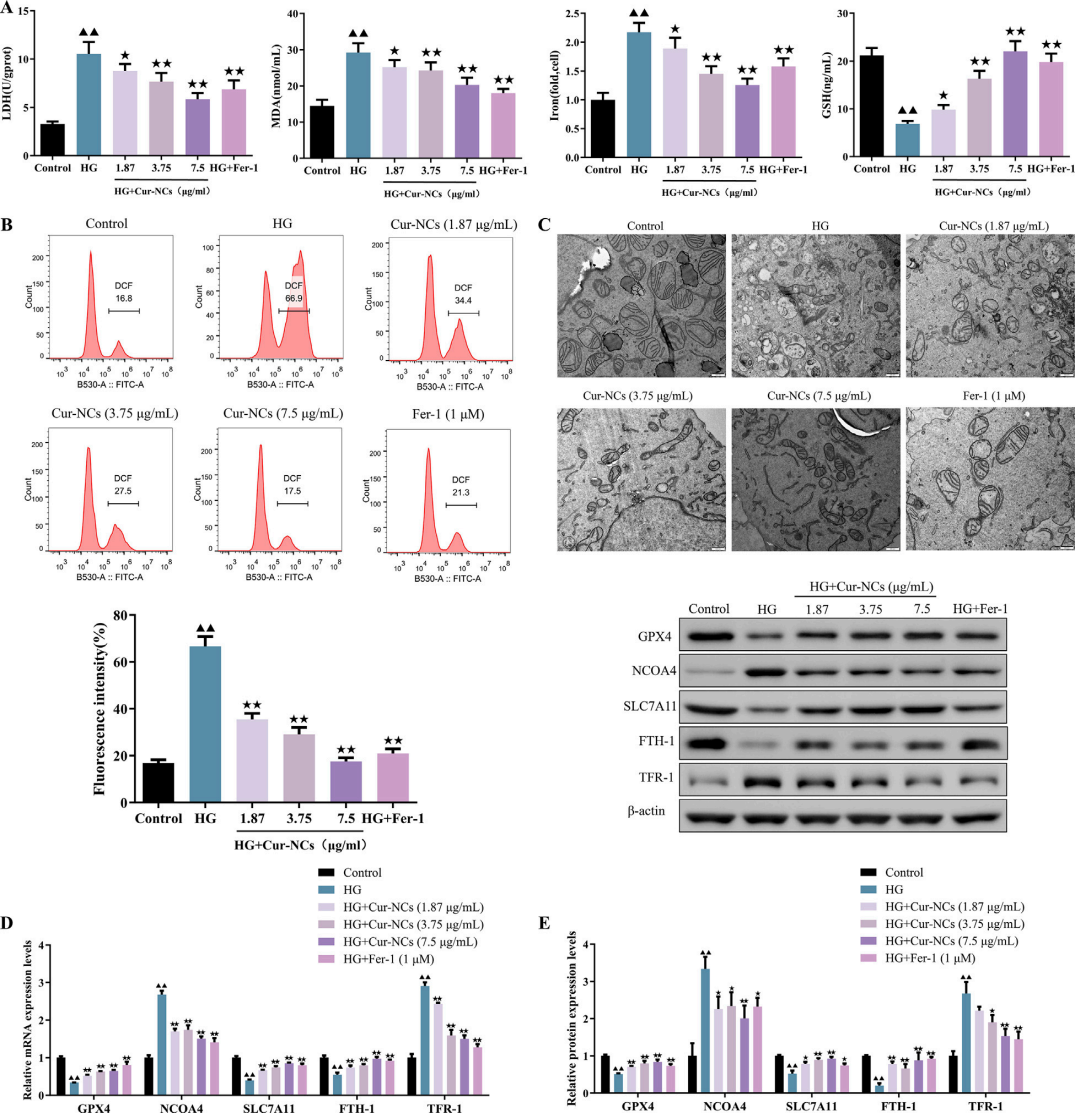
Effect of Cur-NCs on ferroptosis of HG-induced HK-2 cells. **(A)** LDH, MDA, Iron, and GSH levels in HG-induced HK-2 cells were measured by ELISA. **(B)** Lipid ROS levels of Cur-NCs treated HG-induced HK-2 cells were assayed by flow cytometry. **(C)** Representative images of mitochondria in Cur-NCs treated HG-induced HK-2 cells. scale bar, 500 nm. The expression levels of GPX4, NCOA4, SLC7A11, FTH-1, and TFR-1 in Cur-NCs treated HG-induced HK-2 cells were measured by **(D)** qPCR and **(E)** Western blot respectively. ^▲▲^ *p <* 0.01 vs. the control group; ^★^ *p <,* 0.05 ^★★^ *p <* 0.01 vs. the HG group.

To investigate the role of Cur-NCs in HG-induced oxidative damage, HK-2 cells were treated with 30 mM glucose to establish a cell model. Then, the cell death was detected using LDH release. We found that the LDH levels of the HG group increased when compared with the group control, suggesting that the HG condition induced the damage of HK-2. Furthermore, Cur-NCs exhibited a dose-dependent reduction in LDH levels (Figure 2A). As we know, ferroptosis is involved in the death of renal tubular cells in DN (Li et al., 2023a; Li et al., 2023b). Then, the levels of MDA, Iron, GSH and ROS were determined in HG-induced HK-2 cells treated with Cur-NCs. The results showed that the levels of MDA, Iron, and ROS in the different concentrations of Cur-NCs were significantly decreased from that of the HG group, while there was an increase of GSH levels in the Cur-NCs and Fer-1 groups (Figures 2A, B). Importantly, electron microscopic observation revealed that HG-induced mitochondrial damage of ferroptosis in HK-2 cells was characterized by the disappearance of mitochondrial cristae and rupture of the mitochondrial membrane, which was significantly attenuated by Cur-NCs and Fer-1 treatment (Figure 2C). In addition, the expression of ferroptosis-related markers GPX4, NCOA4, SLC7A11, FTH-1 and TFR-1 was detected by Western blotting. The results showed that HG treatment induced ferroptosis, as evidenced by increased expression of NCOA4 and TFR-1, and decreased expression of GPX4, SLC7A11, and FTH-1, which were reversed by Cur-NCs and Fer-1 treatment (Figures 2D, E).

### Cur-NCs evoked its functions through the inhibition of ferroptosis by targeting GPX4

**FIGURE 3:**
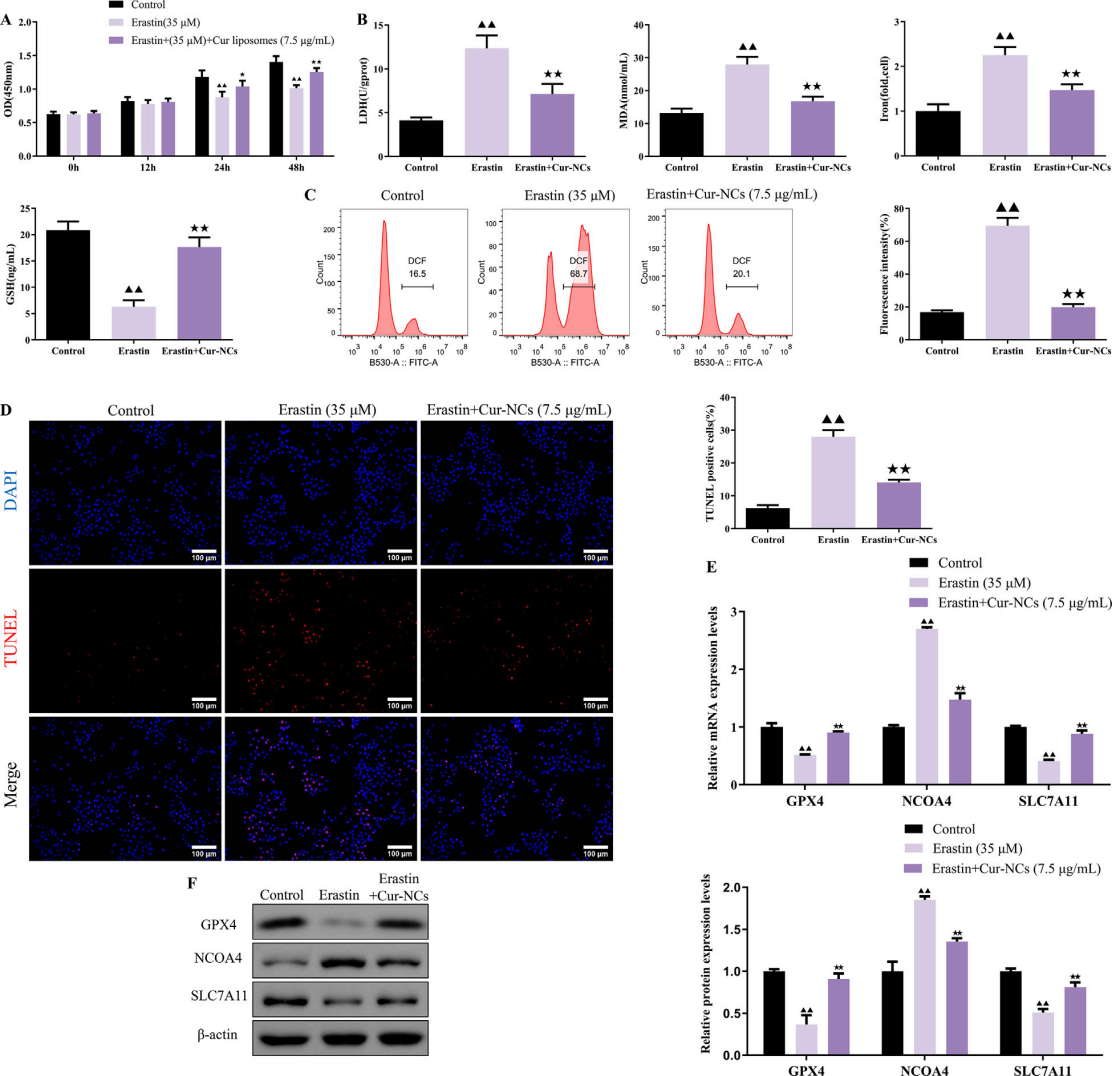
Cur-NCs exert their effects by inhibiting ferroptosis. HK-2 cells were subjected to erastin (35 μM) and/or Cur-NCs (7.5 μg/mL). Next, **(A)** the cell viability, **(B)** LDH, MDA, Iron, GSH, **(C)** ROS, **(D)** cell apoptosis, as well as **(E, F)** GPX4, NCOA4, and SLC7A11 expression levels were detected. ^▲▲^ *p < 0.01* vs. the control group; ^★★^ *p < 0.01* vs. the Erastin group.

**FIGURE 4:**
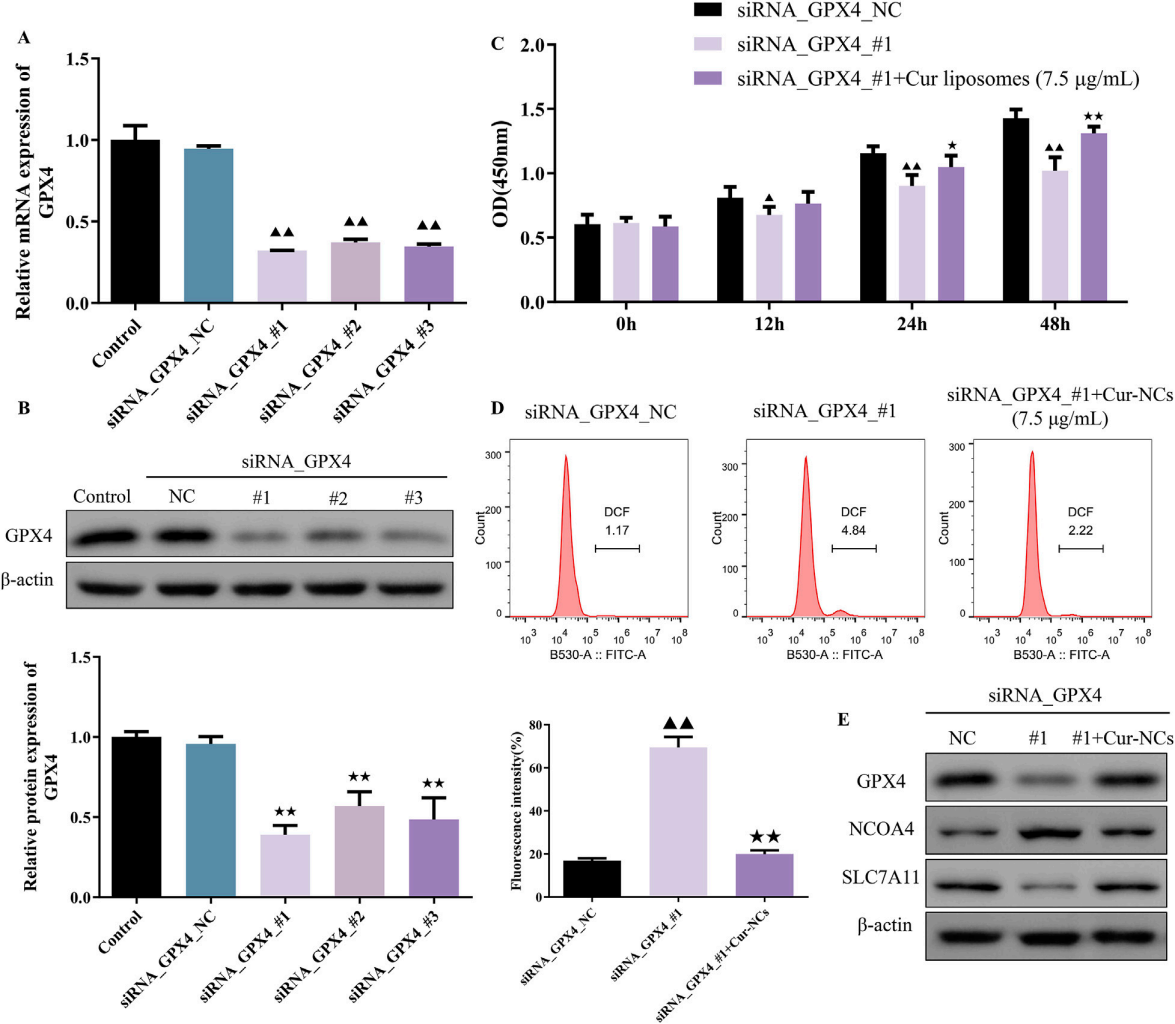
Inhibition of ferroptosis induced by the Cur-NCs was dependent on GPX4. **(A)** qRT-PCR and **(B)** Western blot assays revealed that silencing GPX4 resulted in decreased expression of GPX4 in HK-2 cells. **(C)** A CCK-8 and **(D)** flow cytometry assays showed that Cur-NCs reversed the effects of GPX4 knock-down on cell viability and lipid ROS levels. **(E)** The effects of siRNA_GPX4 or combined with Cur-NCs on GPX4, NCOA4, and SLC7A11 protein expression in HK-2 cells. ^▲▲^ *p <* 0.01 vs. the siRNA_GPX4_NC group; ^★★^ *p <* 0.01 vs. the siRNA_GPX4_#1 group.

**FIGURE 5:**
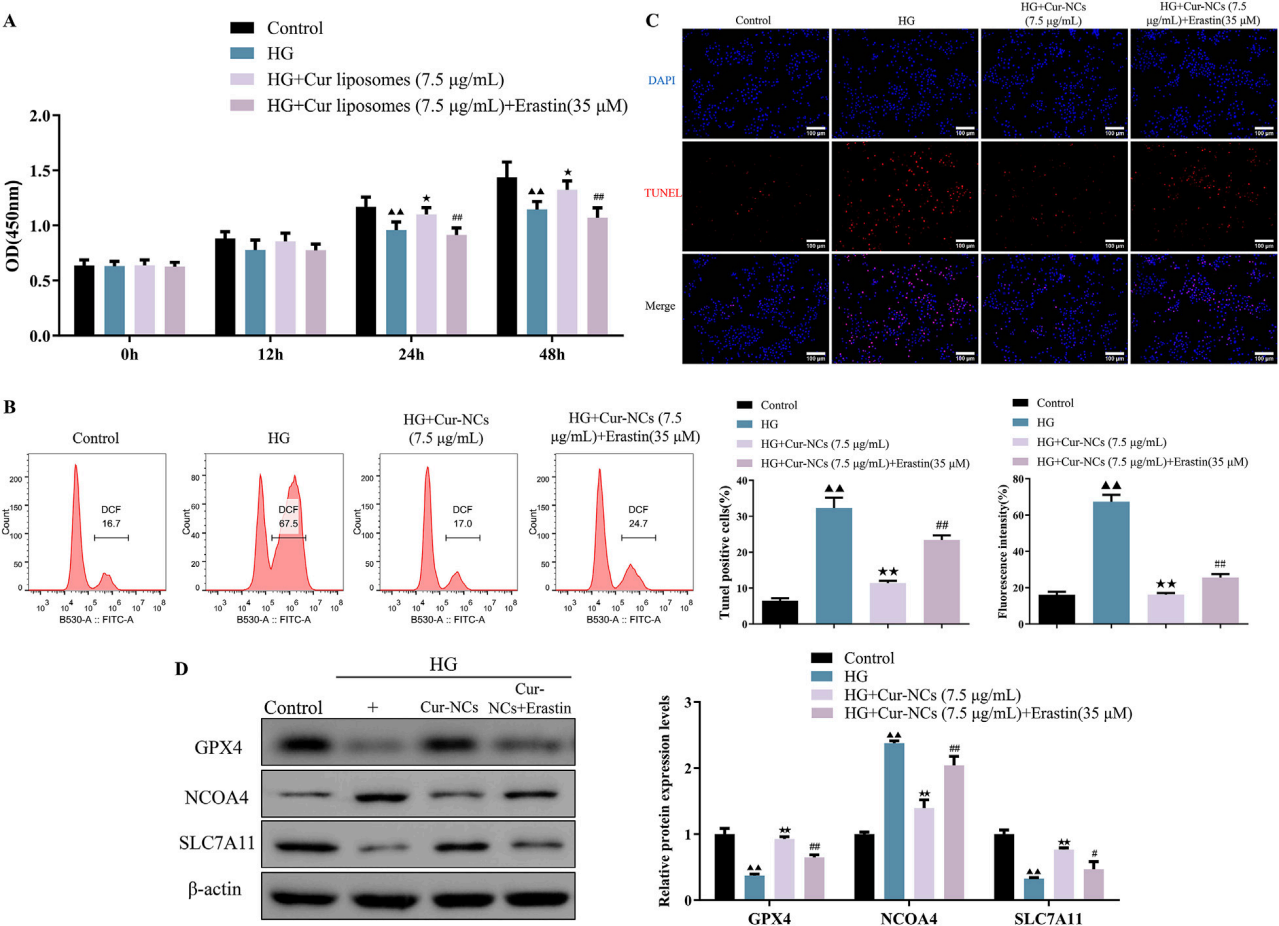
Cur-NCs increase cell viability and relieve apoptosis in HG-induced HK-2 cells via restraining ferroptosis. The cell viability, **(A)** lipid ROS levels, **(B, C)** cell apoptosis levels, as well as **(D)** GPX4, NCOA4, and SLC7A11 protein expression levels in HG-induced HK-2 cells treated with Cur-NCs (7.5 μg/mL) and/or erastin (35 μM) were measured by using CCK-8, flow cytometry, Tunel, and Western blot assays. ^▲▲^ *p <* 0.01 vs. the Control group; ^★^ *p <* 0.05, ^★★^ *p <* 0.01 vs. the HG group; ^#^ *p <* 0.05, ^##^ *p <* 0.01 vs. the HG + Cur-NCs (7.5 μg/mL) group.

To investigate the role of ferroptosis in Cur-NCs’ function, HK-2 cells were treated with erastin (35 μM), a ferroptosis inducer, and/or Cur-NCs (7.5 μg/mL). Compared to the control, erastin dramatically increased LDH, MDA, Iron, ROS, and apoptosis levels while decreasing cell viability and GSH content in HK-2 cells, but Cur-NCs reversed the alterations (Figures 3A–D). In addition, erastin therapy drastically reduced the mRNA and protein expression levels of GPX4 and SLC7A11, although Cur-NCs treatment restored them. Interestingly, Cur-NCs treatment considerably reduced NCOA4 levels (Figures 3E, F). To further identify the potential mechanism of Cur-NCs, the expression levels of GPX4 were markedly decreased in HK-2 cells after transfection with si-GPX4 (Figures 4A, B). In addition, GPX4 knockdown significantly reduced the viability of the HK-2 cells, increased the formation of lipid ROS and NCOA4 expression, and reduced the expression of SLC7A11. However, treatment with Cur-NCs nearly eliminated this effect in the HK-2 cells (Figures 4C–E). Moreover, erastin process reversed the Cur-NCs treatment’s improvement in cell viability as well as its suppression of cell apoptosis and ROS generation in HG-induced HK-2 cells (Figures 5A–C). To verify whether curcumin could inhibit HG-induced ferroptosis in HK-2 cells, we detected the expression of ferroptosis regulatory proteins GPX4, NCOA4 and SLC7A11 by Western blotting. The experimental results showed that the protein expression levels of GPX4 and SLC7A11 were significantly higher and that of NCOA4 was significantly lower in the Cur-NCs-treated group compared with the high-glucose group. However, after adding Erastin for intervention, we observed that the above protective effects of Cur-NCs were significantly suppressed (Figure 5D). Therefore, the results showed that Cur-NCs had the potential to suppress ferroptosis in HG-induced HK-2 cells through the activation of GPX4.

### Cur-NCs ameliorated the hyperlipidemia and renal function in a rat DN model

**FIGURE 6:**
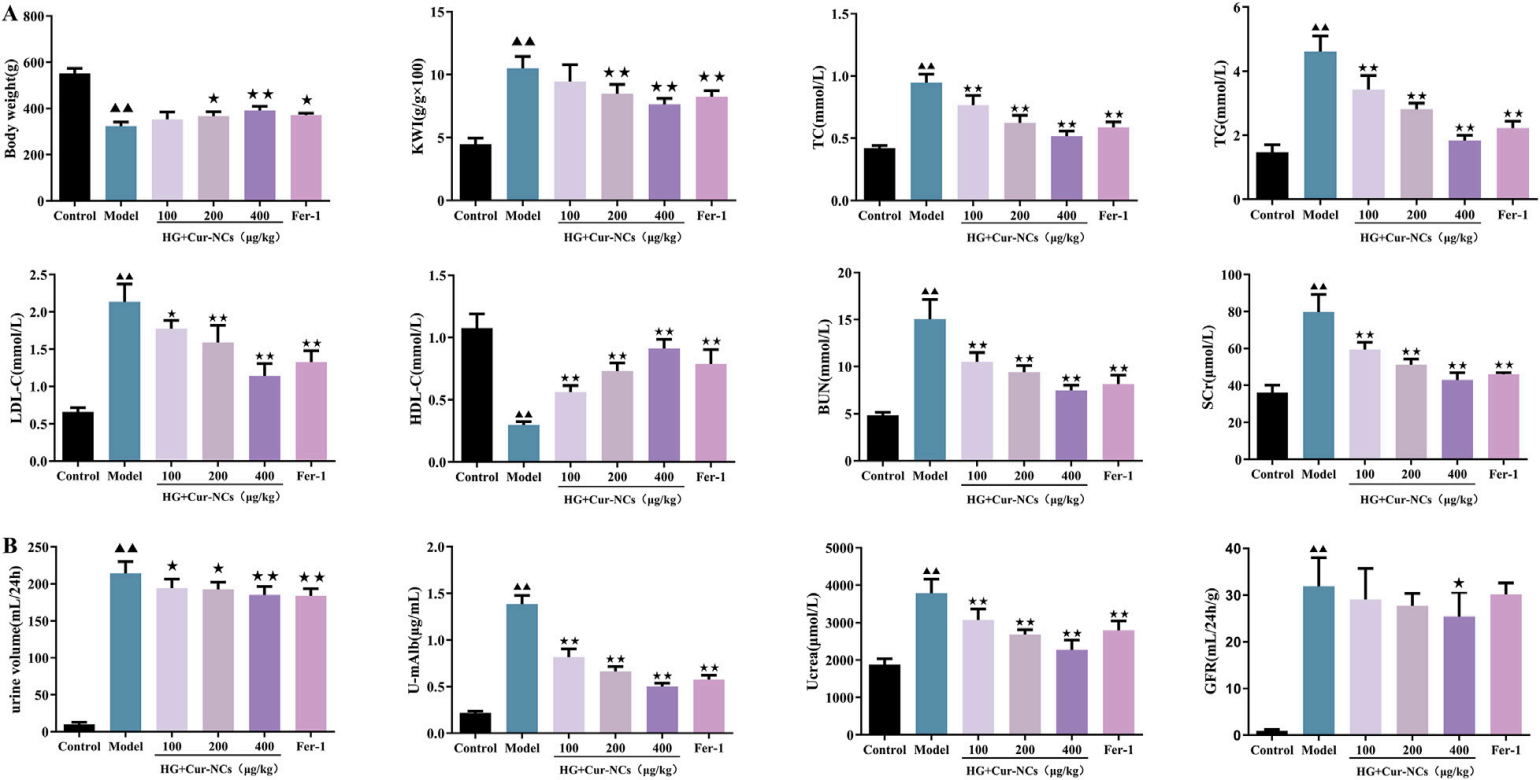
Cur-NCs ameliorated lipid metabolism and kidney function in DN Rats. **(A)** Effects of Cur-NCs on body weight, KWI, serum TC, TG, LDL-C, HDL-C, BUN and SCr in DN Rats were analyzed by using an automatic biochemical analyzer. **(B)** Effects of Cur-NCs on urine volume, U-mAlb, Ucrea and GFR levels in DN Rats. All data are represented as the mean ± SD (n = 6). ^▲▲^ *p <* 0.01 vs. the Control group; ^★^ *p <* 0.05, ^★★^ *p <* 0.01 vs. the Model group.

To explore the protective roles of Cur-NCs in DN *in vivo*, the body weight, KWI, serum TC, TG, LDL-c, HDL-C, BUN, SCr, as well as the urine volume, U-mAlb, Ucrea and GFR were recorded and detected. The results showed that administration with Cur-NCs (100, 200, 400 μg/kg) or Fer-1 significantly increased the body weight and resulted in a significant decrease in KWI, serum TC, TG, LDL-C, HDL-C, BUN, SCr, urine volume, U-mAlb, Ucrea and GFR levels when compared with model group (Figures 6A, B). Notably, the Ucrea and GFR levels in the model group were higher than those in the control group. It suggests that the kidneys were still in the state of ultrafiltration at this stage. In conclusion, these results suggested that Cur-NCs could ameliorate hyperlipidemia and renal function in STZ-induced DN rats.

### Cur-NCs improved oxidative stress and glomerular structure in a rat DN model

**FIGURE 7:**
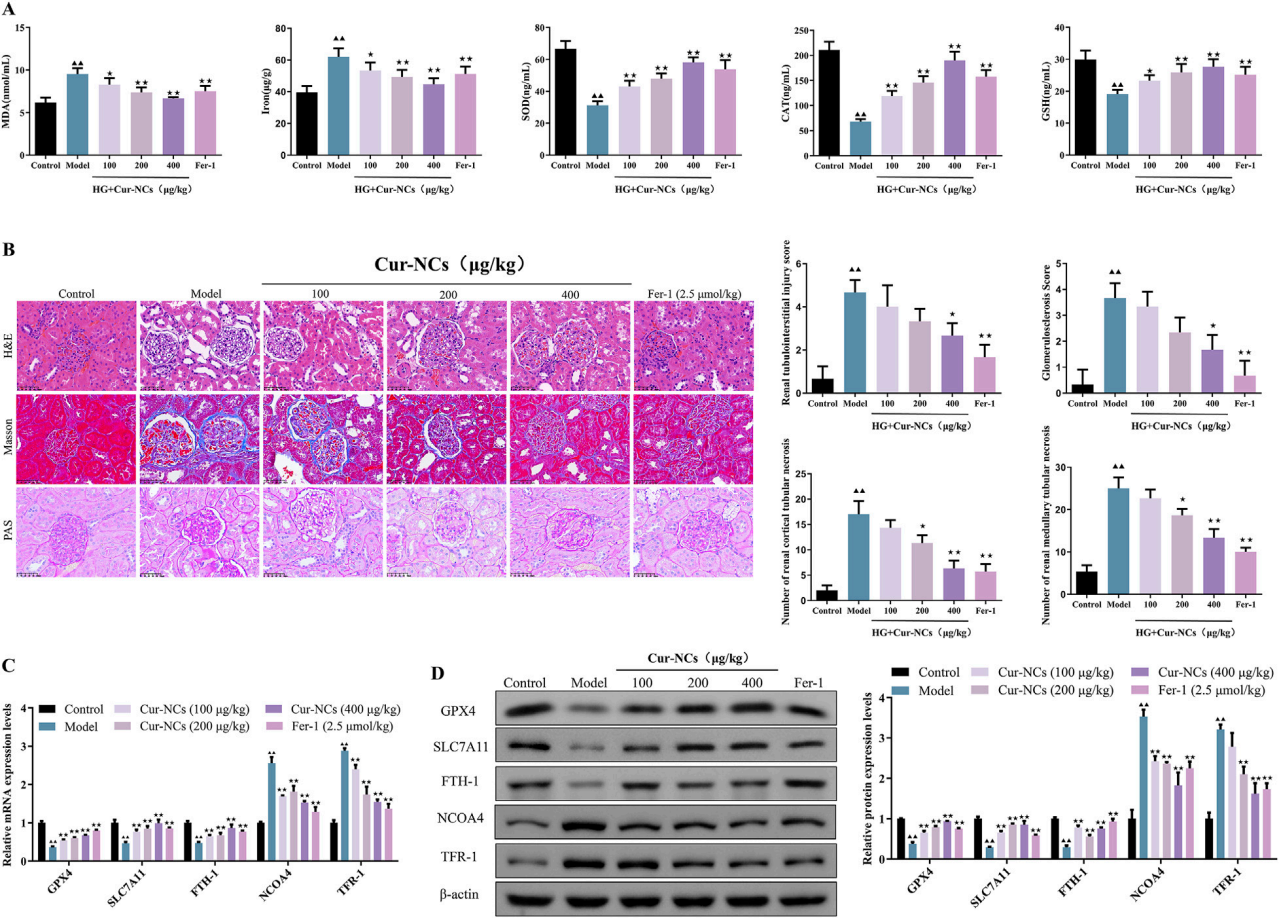
Cur-NCs inhibit oxidative stress and ferroptosis in DN Rats. **(A)** Contents of MDA, Iron, SOD, Cat, and GSH in kidney tissues. **(B)** Representative images of H&E (×400), Masson (×400), and PAS (×400) stained sections of kidney tissues. Scale bar, 50 μm. Included are evaluations of tubulointerstitial and glomerular lesion severity, as well as counts of cellular necrosis tubules in renal cortices and outer medulla. **(C)** mRNA and protein **(D)** levels of GPX4, SLC7A11, FTH-1, NCOA4, and TFR-1 were measured by using qRT-PCR and Western blotting, respectively. All data are represented as the mean ± SD (n = 6). ^▲▲^ *p <* 0.01 vs. the Control group; ^★^ *p <* 0.05, ^★★^ *p <* 0.01 vs. the Model group.

It is generally acknowledged that iron buildup and oxidative stress are important factors in the etiology of ferroptosis in DN. Therefore, we looked at how Cur-NCs affected iron accumulation in the renal tissues of DN rats as well as oxidative stress markers (MDA, SOD, Cat, and GSH). Our findings demonstrated that, following treatment with Cur-NCs or Fer-1, MDA was considerably decreased in the renal tissues of DN rats, whereas SOD, Cat, and GSH production increased (Figure 7A), indicating a decrease in oxidative stress. Furthermore, we saw a decrease in iron accumulation, which adds more credence to Cur-NCs’ antioxidant and iron-regulating abilities. Since renal structural abnormalities are the main cause of renal insufficiency, we evaluated the renal microstructure using H&E, PAS and Masson staining. In control rats, no signs of glomerulosclerosis, interstitial hyperplasia, or basement membrane thickening were observed. In contrast, DN rats exhibited marked dilatation of the thylakoid matrix and tubulointerstitial fibrosis. However, the kidneys of DN rats treated with Cur-NCs or Fer-1 showed a trend toward normalization. To quantify the extent of renal injury, we assessed the severity of tubulointerstitial and glomerular lesions and counted the number of tubules with cellular necrosis in the renal cortex and outer medulla. Our findings revealed that Cur-NC treatment dramatically decreased the severity of tubulointerstitial and glomerular lesions while decreasing the number of necrotic tubules in the renal cortex and outer medulla when compared to untreated DN rats (Figure 7B). In addition, Western blotting results confirmed that Cur-NCs upregulated the protein levels of GPX4, SLC7A11, and FTH-1, while suppressing the expression of NCOA4 and TFR-1 compared with the model group (Figures 7C, D). These findings suggest that Cur-NCs regulate ferritin-related proteins and iron homeostasis, thereby attenuating DN-induced kidney injury.

## Discussion

DN is a severe complication of diabetes mellitus and a leading cause of end-stage renal disease worldwide (Jin et al., 2023; Zhao et al., 2023). Despite extensive research, effective therapeutic strategies for DN remain limited. In this study, we investigated the potential of Cur-NCs to improve DN by inhibiting ferroptosis, a newly recognized form of regulated cell death associated with oxidative stress and inflammation. The results of our study revealed that Cur-NCs significantly ameliorated the pathological manifestations of DN in animal models. This included improvements in renal function and structural preservation of renal tissue. Importantly, we elucidated the molecular mechanism underlying these effects, showing that Cur-NCs targeted GPX4, a key regulator of ferroptosis (Xu et al., 2024a), thereby attenuating oxidative stress in HG-induced HK-2 cells and renal tissues and consequently mitigating DN progression.

Research has shown that the overproduction of lipid peroxides can lead to cell death via ferroptosis (Jiang et al., 2021). Ferroptosis is closely associated with the development of DN (Li et al., 2023b; Mengstie et al., 2023). Ferroptosis is primarily related to iron metabolism disorders, GPX4 antioxidation, and lipid peroxidation (Liu et al., 2022). Under physiological conditions, Fe^3+^ enters cells via transferrin (TF) and is reduced to Fe^2+^ by STEAP3. Fe^2+^ can bind to ferritin heavy chain 1 (FTH1) for storage in cells or be oxidized to Fe^3+^ and transported out of cells via membrane transferrin receptor 1 (TFR1), participating in the body’s iron circulation to maintain iron homeostasis (Dong et al., 2020; Plays et al., 2021). When iron is overloaded, high concentrations of Fe^2+^ undergo the Fenton reaction, generating ROS that cause oxidative damage to cell membranes, ultimately resulting in cell death via ferroptosis (Zhao et al., 2021). Our study found that the levels of Fe in HG-induced HK-2 cells and kidney tissues of DN rats were significantly higher than those in the control group, which may be associated with a significant decrease in FTH1 levels. However, intervention with Cur-NCs alleviated iron-mediated cell death in HG-induced HK-2 cells and kidney tissues of DN rats in this study. GPX4 plays a crucial role in cellular defense against oxidative stress by catalyzing the reduction of lipid hydroperoxides to their corresponding alcohols. Its importance in kidney physiology and pathology has been increasingly recognized. Studies have demonstrated that GPX4 deficiency exacerbates renal injury in various experimental models, highlighting its protective role against oxidative damage and inflammation in the kidney (Hosohata et al., 2022; Li et al., 2022b). Our findings add to this body of knowledge by showing that modulation of GPX4 activity with Cur-NCs can mitigate DN progression by inhibiting ferroptosis.

Furthermore, our study underscores the potential of nanotechnology in drug therapy. Nanocrystals possess advantages such as a large specific surface area, rapid dissolution rate, and high drug-loading capacity, which can enhance the bioavailability of drugs and improve therapeutic efficacy. The practical value of nanocrystals is demonstrated by the fact that multiple nanocrystal-based drugs are already in clinical use. Therefore, the development of Cur-NCs holds promise as an effective treatment for DN by modulating GPX4 activity. Despite the encouraging results of our study, several limitations warrant consideration. At present, our research has primarily focused on exploring the potential of Cur-NCs using cell and animal models. However, we acknowledge that a rigorous preclinical evaluation of Cur-NCs, including safety assessments such as acute and potential long-term toxicity studies, as well as pharmacodynamic investigations across various animal species, has yet to be conducted. This comprehensive preclinical assessment represents a critical next step in our research, and we plan to undertake it in the future with the aim of laying a solid foundation for eventual clinical trials in human subjects, which are essential for validating the safety and efficacy of Cur-NCs in the treatment of DN. Additionally, while we identified GPX4 as a key mediator of Cur-NCs effects in DN, the precise molecular mechanisms underlying GPX4 modulation and ferroptosis inhibition require further investigation. Finally, the complexity of DN pathogenesis suggests that a multifaceted approach combining various therapeutic strategies may be necessary to fully address the disease’s progression.

## Conclusion

In conclusion, the current study provided compelling evidence that Cur-NCs are effective in slowing the progression of DN by inhibiting ferroptosis. This therapeutic effect was achieved through the upregulation of GPX4, a key regulator of ferroptosis, which attenuated oxidative stress and protected renal tissues from further damage in a cellular model of renal tubular injury and an animal model of DN. These findings highlight the potential application of Cur-NCs in the treatment of DN, highlighting the great potential of nanotechnology in drug therapy.

## Data Availability

The datasets presented in this study can be found in online repositories. The names of the repository/repositories and accession number(s) can be found below: https://figshare.com/, https://doi.org/10.6084/m9.figshare.27193149.v2.

## Supplementary material

SUPPLEMENTARY FIGURE S1 Establishment of STZ induced diabetes model in SD rats.

## References

[B1] Ahmed S. A. Aziz W. M. Shaker S. E. Fayed D. B. Shawky H. (2022). Urinary transferrin and proinflammatory markers predict the earliest diabetic nephropathy onset. Biomarkers 27, 178–187. 10.1080/1354750X.2021.2023639 34957874

[B2] ALTamimi J. Z. Alfaris N. A. Al-Farga A. M. Alshammari G. M. Binmowyna M. N. Yahya M. A. (2021). Curcumin reverses diabetic nephropathy in streptozotocin-induced diabetes in rats by inhibition of PKCβ/p66Shc axis and activation of FOXO-3a. J. Nutr. Biochem. 87, 108515. 10.1016/j.jnutbio.2020.108515 33017608

[B3] Chen J. Liu Q. He J. Li Y. (2022a). Immune responses in diabetic nephropathy: pathogenic mechanisms and therapeutic target. Front. Immunol. 13, 958790. 10.3389/fimmu.2022.958790 36045667 PMC9420855

[B4] Chen J. Ou Z. Gao T. Yang Y. Shu A. Xu H. (2022b). Ginkgolide B alleviates oxidative stress and ferroptosis by inhibiting GPX4 ubiquitination to improve diabetic nephropathy. Biomed. Pharmacother. 156, 113953. 10.1016/j.biopha.2022.113953 36411664

[B5] Chen M. M. Jia J. H. Tan Y. J. Ren Y. S. Lv J. L. Chu T. (2023). Shen-Qi-Jiang-Tang granule ameliorates diabetic nephropathy via modulating tumor necrosis factor signaling pathway. J. Ethnopharmacol. 303, 116031. 10.1016/j.jep.2022.116031 36503032

[B6] Dong H. Qiang Z. Chai D. Peng J. Xia Y. Hu R. (2020). Nrf2 inhibits ferroptosis and protects against acute lung injury due to intestinal ischemia reperfusion via regulating SLC7A11 and HO-1. Aging (Albany NY) 12, 12943–12959. 10.18632/aging.103378 32601262 PMC7377827

[B7] Feng Q. Yang Y. Ren K. Qiao Y. Sun Z. Pan S. (2023). Broadening horizons: the multifaceted functions of ferroptosis in kidney diseases. Int. J. Biol. Sci. 19, 3726–3743. 10.7150/ijbs.85674 37564215 PMC10411478

[B8] Ganugula R. Nuthalapati N. K. Dwivedi S. Zou D. Arora M. Friend R. (2023). Nanocurcumin combined with insulin alleviates diabetic kidney disease through P38/P53 signaling axis. J. Control Release 353, 621–633. 10.1016/j.jconrel.2022.12.012 36503070 PMC9904426

[B9] Gao N. Zhang Y. Li L. Lei L. Cao P. Zhao X. (2020). Hyperhomocysteinemia-induced oxidative stress aggravates renal damage in hypertensive rats. Am. J. Hypertens. 33, 1127–1135. 10.1093/ajh/hpaa086 32484231

[B10] Hosohata K. Harnsirikarn T. Chokesuwattanaskul S. (2022). Ferroptosis: a potential therapeutic target in acute kidney injury. Int. J. Mol. Sci. 23, 6583. 10.3390/ijms23126583 35743026 PMC9223765

[B11] Hu Q. Chen Y. Deng X. Li Y. Ma X. Zeng J. (2023). Diabetic nephropathy: focusing on pathological signals, clinical treatment, and dietary regulation. Biomed. Pharmacother. 159, 114252. 10.1016/j.biopha.2023.114252 36641921

[B12] Huang D. Shen P. Wang C. Gao J. Ye C. Wu F. (2022). Calycosin plays a protective role in diabetic kidney disease through the regulation of ferroptosis. Pharm. Biol. 60, 990–996. 10.1080/13880209.2022.2067572 35587919 PMC9132481

[B13] Huang S. Xu D. Zhang L. Hao L. Jia Y. Zhang X. (2023). Therapeutic effects of curcumin liposomes and nanocrystals on inflammatory osteolysis: *in vitro* and *in vivo* comparative study. Pharmacol. Res. 192, 106778. 10.1016/j.phrs.2023.106778 37094714

[B14] Hussain Y. Khan H. Alotaibi G. Khan F. Alam W. Aschner M. (2022). How curcumin targets inflammatory mediators in diabetes: therapeutic insights and possible solutions. Molecules 27, 4058. 10.3390/molecules27134058 35807304 PMC9268477

[B15] Jiang X. Stockwell B. R. Conrad M. (2021). Ferroptosis: mechanisms, biology and role in disease. Nat. Rev. Mol. Cell Biol. 22, 266–282. 10.1038/s41580-020-00324-8 33495651 PMC8142022

[B16] Jin Q. Liu T. Qiao Y. Liu D. Yang L. Mao H. (2023). Oxidative stress and inflammation in diabetic nephropathy: role of polyphenols. Front. Immunol. 14, 1185317. 10.3389/fimmu.2023.1185317 37545494 PMC10401049

[B17] Li J. Li L. Zhang Z. Chen P. Shu H. Yang C. (2023a). Ferroptosis: an important player in the inflammatory response in diabetic nephropathy. Front. Immunol. 14, 1294317. 10.3389/fimmu.2023.1294317 38111578 PMC10725962

[B18] Li L. Dai Y. Ke D. Liu J. Chen P. Wei D. (2023b). Ferroptosis: new insight into the mechanisms of diabetic nephropathy and retinopathy. Front. Endocrinol. (Lausanne) 14, 1215292. 10.3389/fendo.2023.1215292 37600716 PMC10435881

[B19] Li Q. Liao J. Chen W. Zhang K. Li H. Ma F. (2022a). NAC alleviative ferroptosis in diabetic nephropathy via maintaining mitochondrial redox homeostasis through activating SIRT3-SOD2/Gpx4 pathway. Free Radic. Biol. Med. 187, 158–170. 10.1016/j.freeradbiomed.2022.05.024 35660452

[B20] Li Z. Zhu Z. Liu Y. Liu Y. Zhao H. (2022b). Function and regulation of GPX4 in the development and progression of fibrotic disease. J. Cell Physiol. 237, 2808–2824. 10.1002/jcp.30780 35605092

[B21] Liang D. Liu L. Qi Y. Nan F. Huang J. Tang S. (2024). Jin-Gui-Shen-Qi Wan alleviates fibrosis in mouse diabetic nephropathy via MHC class II. J. Ethnopharmacol. 324, 117745. 10.1016/j.jep.2024.117745 38228231

[B22] Lin Y. C. Chang Y. H. Yang S. Y. Wu K. D. Chu T. S. (2018). Update of pathophysiology and management of diabetic kidney disease. J. Formos. Med. Assoc. 117, 662–675. 10.1016/j.jfma.2018.02.007 29486908

[B23] Liu J. Kang R. Tang D. (2022). Signaling pathways and defense mechanisms of ferroptosis. Febs J. 289, 7038–7050. 10.1111/febs.16059 34092035

[B24] Machado D. I. De Oliveira Silva E. Ventura S. Vattimo M. F. F. (2022). The effect of curcumin on renal ischemia/reperfusion injury in diabetic rats. Nutrients 14, 2798. 10.3390/nu14142798 35889755 PMC9323852

[B25] Mengstie M. A. Seid M. A. Gebeyehu N. A. Adella G. A. Kassie G. A. Bayih W. A. (2023). Ferroptosis in diabetic nephropathy: mechanisms and therapeutic implications. Metabol. Open 18, 100243. 10.1016/j.metop.2023.100243 37124126 PMC10130620

[B26] Noshahr Z. S. Salmani H. Khajavi Rad A. Sahebkar A. (2020). Animal models of diabetes-associated renal injury. J. Diabetes Res. 2020, 9416419. 10.1155/2020/9416419 32566684 PMC7256713

[B27] Plays M. Müller S. Rodriguez R. (2021). Chemistry and biology of ferritin. Metallomics 13, mfab021. 10.1093/mtomcs/mfab021 33881539 PMC8083198

[B28] Samsu N. (2021). Diabetic nephropathy: challenges in pathogenesis, diagnosis, and treatment. Biomed. Res. Int. 2021, 1497449. 10.1155/2021/1497449 34307650 PMC8285185

[B29] Selby N. M. Taal M. W. (2020). An updated overview of diabetic nephropathy: diagnosis, prognosis, treatment goals and latest guidelines. Diabetes Obes. Metab. 22 (Suppl. 1), 3–15. 10.1111/dom.14007 32267079

[B30] Shen S. Ji C. Wei K. (2022). Cellular senescence and regulated cell death of tubular epithelial cells in diabetic kidney disease. Front. Endocrinol. (Lausanne) 13, 924299. 10.3389/fendo.2022.924299 35837297 PMC9273736

[B31] Sun H. Saeedi P. Karuranga S. Pinkepank M. Ogurtsova K. Duncan B. B. (2022). IDF Diabetes Atlas: global, regional and country-level diabetes prevalence estimates for 2021 and projections for 2045. Diabetes Res. Clin. Pract. 183, 109119. 10.1016/j.diabres.2021.109119 34879977 PMC11057359

[B32] Tanase D. M. Gosav E. M. Anton M. I. Floria M. Seritean Isac P. N. Hurjui L. L. (2022). Oxidative stress and NRF2/KEAP1/ARE pathway in diabetic kidney disease (DKD): new perspectives. Biomolecules 12, 1227. 10.3390/biom12091227 36139066 PMC9496369

[B33] Tu Q. Li Y. Jin J. Jiang X. Ren Y. He Q. (2019). Curcumin alleviates diabetic nephropathy via inhibiting podocyte mesenchymal transdifferentiation and inducing autophagy in rats and MPC5 cells. Pharm. Biol. 57, 778–786. 10.1080/13880209.2019.1688843 31741405 PMC6882478

[B34] Tuttle K. R. Agarwal R. Alpers C. E. Bakris G. L. Brosius F. C. Kolkhof P. (2022). Molecular mechanisms and therapeutic targets for diabetic kidney disease. Kidney Int. 102, 248–260. 10.1016/j.kint.2022.05.012 35661785

[B35] Valdivielso J. M. Jacobs-Cachá C. Soler M. J. (2019). Sex hormones and their influence on chronic kidney disease. Curr. Opin. Nephrol. Hypertens. 28, 1–9. 10.1097/MNH.0000000000000463 30320621

[B36] Wang C. Han Z. Wu Y. Lu X. Tang X. Xiao J. (2021a). Enhancing stability and anti-inflammatory properties of curcumin in ulcerative colitis therapy using liposomes mediated colon-specific drug delivery system. Food Chem. Toxicol. 151, 112123. 10.1016/j.fct.2021.112123 33744379

[B37] Wang L. Wang H. L. Liu T. T. Lan H. Y. (2021b). TGF-beta as a master regulator of diabetic nephropathy. Int. J. Mol. Sci. 22, 7881. 10.3390/ijms22157881 34360646 PMC8345981

[B38] Wang X. Liao Z. Zhao G. Dong W. Huang X. Zhou X. (2023). Curcumin nanocrystals self-stabilized Pickering emulsion freeze-dried powder: development, characterization, and suppression of airway inflammation. Int. J. Biol. Macromol. 245, 125493. 10.1016/j.ijbiomac.2023.125493 37348593

[B39] Wu Y. Sun Y. Wu Y. Zhang K. Chen Y. (2023). Predictive value of ferroptosis-related biomarkers for diabetic kidney disease: a prospective observational study. Acta Diabetol. 60, 507–516. 10.1007/s00592-022-02028-1 36633709 PMC10033569

[B40] Xie T. Chen X. Chen W. Huang S. Peng X. Tian L. (2021). Curcumin is a potential adjuvant to alleviates diabetic retinal injury via reducing oxidative stress and maintaining Nrf2 pathway homeostasis. Front. Pharmacol. 12, 796565. 10.3389/fphar.2021.796565 34955862 PMC8702852

[B41] Xu X. Xu X. D. Ma M. Q. Liang Y. Cai Y. B. Zhu Z. X. (2024a). The mechanisms of ferroptosis and its role in atherosclerosis. Biomed. Pharmacother. 171, 116112. 10.1016/j.biopha.2023.116112 38171246

[B42] Xu Z. Cai K. Su S. L. Zhu Y. Liu F. Duan J. A. (2024b). Salvianolic acid B and tanshinone IIA synergistically improve early diabetic nephropathy through regulating PI3K/Akt/NF-κB signaling pathway. J. Ethnopharmacol. 319, 117356. 10.1016/j.jep.2023.117356 37890803

[B43] Yang X. Dong X. Li J. Zheng A. Shi W. Shen C. (2024). Nanocurcumin attenuates pyroptosis and inflammation through inhibiting NF-κB/GSDMD signal in high altitude-associated acute liver injury. J. Biochem. Mol. Toxicol. 38, e23606. 10.1002/jbt.23606 38050447

[B44] Yao L. Liang X. Qiao Y. Chen B. Wang P. Liu Z. (2022). Mitochondrial dysfunction in diabetic tubulopathy. Metabolism 131, 155195. 10.1016/j.metabol.2022.155195 35358497

[B45] Zhang P. Fang J. Zhang J. Ding S. Gan D. (2020). Curcumin inhibited podocyte cell apoptosis and accelerated cell autophagy in diabetic nephropathy via regulating Beclin1/UVRAG/Bcl2. Diabetes Metab. Syndr. Obes. 13, 641–652. 10.2147/DMSO.S237451 32184643 PMC7060797

[B46] Zhao H. Yang C. E. Liu T. Zhang M. X. Niu Y. Wang M. (2023). The roles of gut microbiota and its metabolites in diabetic nephropathy. Front. Microbiol. 14, 1207132. 10.3389/fmicb.2023.1207132 37577423 PMC10413983

[B47] Zhao T. Guo X. Sun Y. (2021). Iron accumulation and lipid peroxidation in the aging retina: implication of ferroptosis in age-related macular degeneration. Aging Dis. 12, 529–551. 10.14336/AD.2020.0912 33815881 PMC7990372

[B48] Zhao X. Hui Q. C. Xu R. Gao N. Cao P. (2022). Resveratrol: a new approach to ameliorate hyperhomocysteinaemia-induced renal dysfunction. Exp. Ther. Med. 24, 510. 10.3892/etm.2022.11437 35837032 PMC9257945

[B49] Zheng X. Zhang J. Zhang L. Huangfu X. Li Y. Chen J. (2024). Controlled preparation of curcumin nanocrystals by detachable stainless steel microfluidic chip. Int. J. Pharm. 663, 124574. 10.1016/j.ijpharm.2024.124574 39134290

[B50] Zhu X. Xu X. Du C. Su Y. Yin L. Tan X. (2022). An examination of the protective effects and molecular mechanisms of curcumin, a polyphenol curcuminoid in diabetic nephropathy. Biomed. Pharmacother. 153, 113438. 10.1016/j.biopha.2022.113438 36076553

