# Supplementary material for: Curcumin nanocrystals ameliorate ferroptosis of diabetic nephropathy through glutathione peroxidase 4
Source: Front Pharmacol. 2025 Jan 6;15:1508312. doi: 10.3389/fphar.2024.1508312 (PMC11743454; doi:10.3389/fphar.2024.1508312)
Supplement: Supplementary file 1 [file Table1.docx]

Table S1. Primer Sequences for qRT-PCR

| Gene | Forward Primer | Reverse Primer |
| --- | --- | --- |
| GPX4 | 5′-GTTACTCCCTGGCTCCTG-3′ | 5′-CTCCCAGTGAGGCAAGAC-3′ |
| NCOA4 | 5′-TGATCTCCAACCTTTTCC-3′ | 5′-CTTACATACCCAGCACCG-3′ |
| SLC7A11 | 5′-TCTCCAAAGGAGGTTACCTGC-3′ | 5′-AGACTCCCCTCAGTAAAGTGAC-3′ |
| FTH-1 | 5′-TCCTACGTTTACCTGTCCATGT-3′ | 5′-GTTTGTGCAGTTCCAGTAGTGA-3′ |
| TFR-1 | 5′-ACCATTGTCATATACCCGGTTCA-3′ | 5′-CAATAGCCCAAGTAGCCAATCAT-3′ |
| β-actin | 5′-GATGACCCAGATCATGTTTGAG-3′ | 5′-TAATGTCACGCACGATTTCC-3′ |
