# Supplementary figures and images for: Curcumin nanocrystals ameliorate ferroptosis of diabetic nephropathy through glutathione peroxidase 4
Source: Front Pharmacol. 2025 Jan 6;15:1508312. doi: 10.3389/fphar.2024.1508312 (PMC11743454; doi:10.3389/fphar.2024.1508312)

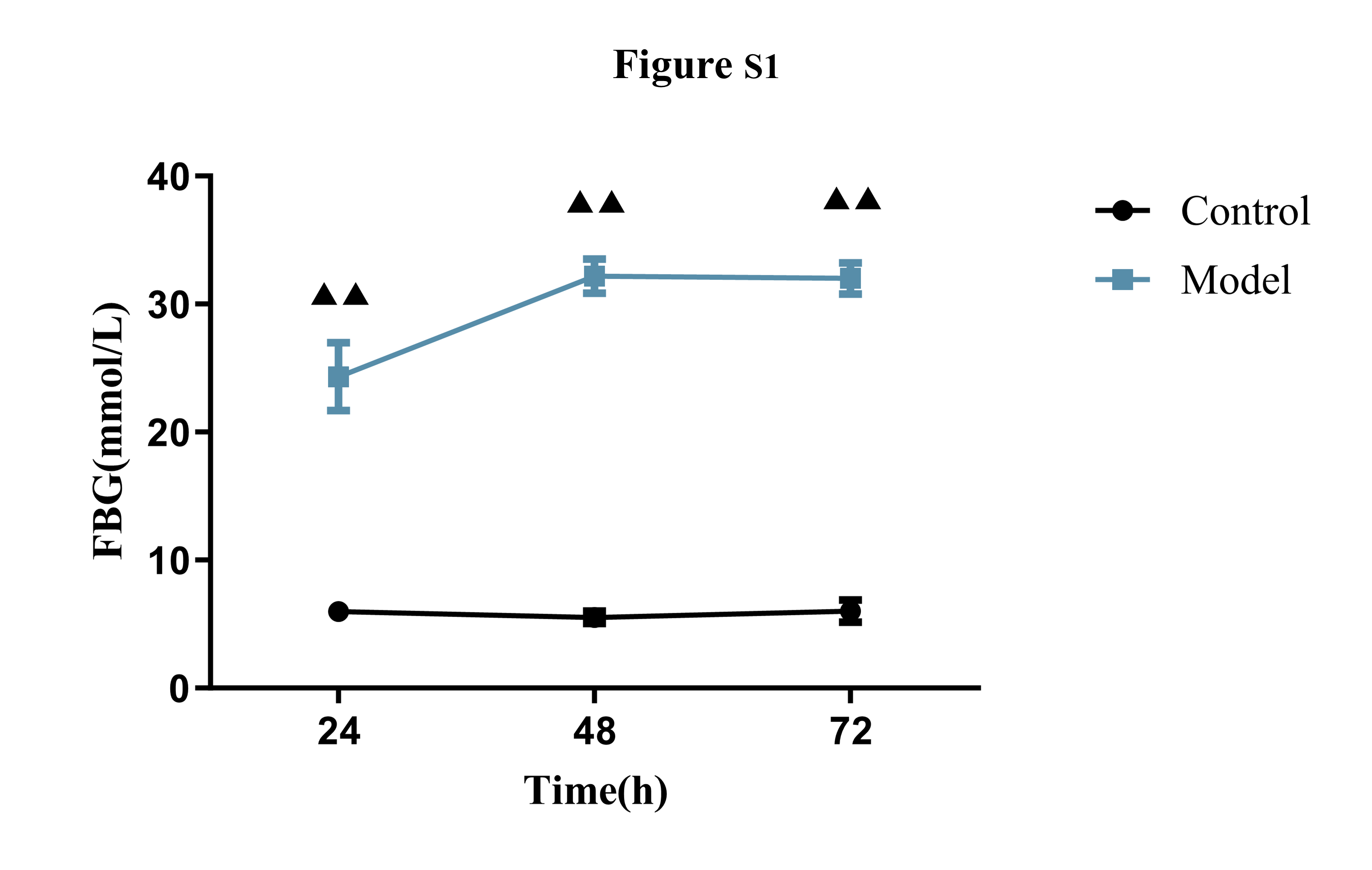

Supplement: Supplementary file 2 [file Image1.tif]
